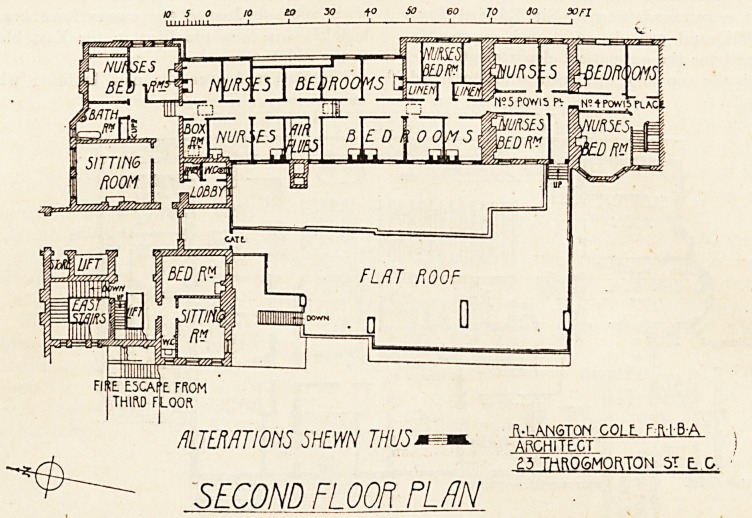# National Hospital for the Paralysed and Epileptic, Queen Square

**Published:** 1910-04-09

**Authors:** 


### NATIONAL HOSPITAL FOR THE PARALYSED AND EPILEPTIC, QUEEN SQUARE.

### THE JUBILEE ALTERATIONS.

The new buildings and alterations shown on the plans we publish to-day have been carried out mainly in compliance with the recommendations of Sir Edward Fry's Committee in 1901, and provide additional accommodation for nurses and for the out-patient department.

Beginning with the top floor, thirteen additional bedrooms for nurses, with a boxroom, linen cupboards, and bathroom have been formed in the roof over Princess Christian ward. An opening made in the wall of No. 5 Powis Place affords access from the corridor of the new rooms to the second floor of Nos. 4 and 5 Powis Place, and so to the staircase in No. 4, which thus provides a second means of escape in case of fire. An additional storey has been built on the Powis Place houses, in which seven more bedrooms for nurses have been formed. A bed lift has been provided in the East block for access to the isolation wards.

The flat roof over the new building which has been erected between Powis Place and Queen Square is to be used as a roof garden for the nurses. The new building, which is of three storeys only, contains on the first floor a dining-room for nurses, with a pantry adjoining, a recreation-room for nurses, and a bathroom and w.c. to serve the bedrooms in the Powis Place houses. Adjoin-

R. LANGTON COLE F.R.I.B.A.

ARCHITECT

23 THROGMORTON St E.C.

Nos 4 & 5 POWIS PLACE

MEZZANINE FLOOR.

ing the dining-room is the assistant matron's bedroom, next to which is a bathroom for the resident medical officer, whose quarters are close by. The new block must to some extent interfere with the light and ventilation of the Princess Christian ward, from which it is distant only some 16 feet.

On the ground floor the alterations comprise an extension of the out-patient waiting hall; two new consulting rooms, each provided with two dressing-rooms; an ophthalmic room; a new and larger dispensary, with a small waiting-room for patients.

The basement has been rearranged, and a new photographic dark-room and testing-room, also store and work rooms for the dispensary, provided. A new mortuary has also been built.

The alterations have been carried out from the designs and under the supervision of Mr. R. Langton Cole, F.R.I.B.A., the architect to the hospital.

SECOND FLOOR PLAN

## Figures and Tables

**Figure f1:**
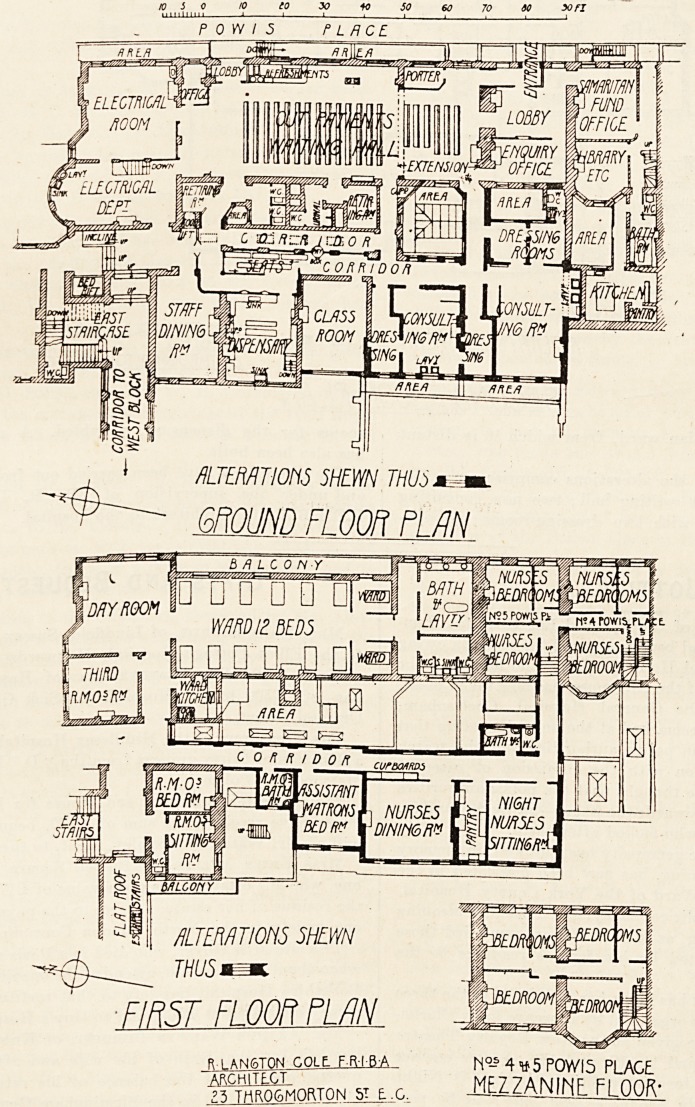


**Figure f2:**